# Adenosine A_1_ receptor signaling inhibits BK channels through a PKCα-dependent mechanism in mouse aortic smooth muscle

**DOI:** 10.1002/phy2.37

**Published:** 2013-07-29

**Authors:** S S Kunduri, G M Dick, M A Nayeem, S J Mustafa

**Affiliations:** 1 Department of Physiology and Pharmacology, West Virginia UniversityMorgantown, West Virginia, 26506; 2 Center for Cardiovascular and Respiratory Sciences, West Virginia UniversityMorgantown, West Virginia, 26506; 3 Department of Exercise Physiology, West Virginia University School of MedicineMorgantown, West Virginia, 26506

**Keywords:** 20-hydroxy-eicosatetraenoic acid, 2-chloro-N (6)-cyclopentyladenosine, 5′-N-ethylcarboxamidoadenosine, Large conductance Ca^2+^/voltage-sensitive K^+^ channels, protein kinase C alpha

## Abstract

Adenosine receptors (AR; A_1_, A_2A_, A_2B_, and A_3_) contract and relax smooth muscle through different signaling mechanisms. Deciphering these complex responses remains difficult because relationships between AR subtypes and various end-effectors (e.g., enzymes and ion channels) remain to be identified. A_1_AR stimulation is associated with the production of 20–hydroxyeicosatetraenoic acid (20–HETE) and activation of protein kinase C (PKC). 20–HETE and PKC can inhibit large conductance Ca^2+^/voltage-sensitive K^+^ (BK) channels that regulate smooth muscle contraction. We tested the hypothesis that activation of A_1_AR inhibits BK channels via a PKC-dependent mechanism. Patch clamp recordings and Western blots were performed using aortae of wild type (WT) and A_1_AR knockout (A_1_KO) mice. There were no differences in whole-cell K^+^ current or α and β1 subunits expression between WT and A_1_KO. 20–HETE (100 nmol/L) inhibited BK current similarly in WT and A_1_KO mice. NECA (5′–N–ethylcarboxamidoadenosine; 10 μmol/L), a nonselective AR agonist, increased BK current in myocytes from both WT and A_1_KO mice, but the increase was greater in A_1_KO (52 ± 15 vs. 17 ± 3%; *P* < 0.05). This suggests that A_1_AR signaling negatively regulates BK channel activity. Accordingly, CCPA (2–chloro–N(6)-cyclopentyladenosine; 100 nmol/L), an A_1_AR-selective agonist, inhibited BK current in myocytes from WT but not A_1_KO mice (81 ± 4 vs. 100 ± 7% of control; *P* < 0.05). Gö6976 (100 nmol/L), a PKCα inhibitor, abolished the effect of CCPA to inhibit BK current (99 ± 3% of control). These data lead us to conclude that, in aortic smooth muscle, A_1_AR inhibits BK channel activity and that this occurs via a mechanism involving PKCα.

## Introduction

Adenosine exerts its effects through four G-protein coupled receptors: the known adenosine receptor (AR) subtypes are A_1_, A_2A_, A_2B_, and A_3_. These AR subtypes play important roles in vascular reactivity, as A_1_AR and A_3_AR contract smooth muscle, whereas A_2A_AR and A_2B_AR relax smooth muscle (Fredholm 2001; Tawfik et al. 2005; Jacobson and Gao 2006; Ansari et al. 2007; Ponnoth et al. 2009). It is well accepted that metabolites of arachidonic acid (AA) regulate vascular tone; however, only recently have these pathways been recognized to function downstream of A_1_AR and A_2A_AR (Harder et al. 1997; Cheng et al. 2004; Nayeem et al. 2008; Ponnoth et al. 2012b). Epoxyeicosatrienoic acids (EETs) and 20-hydroxyeicosatetraenoic acid (20-HETE) are produced from arachidonate by epoxygenases and ω-hydroxylases, respectively. EETs are considered to be endothelium-derived hyperpolarizing factors that activate Ca^2+^-dependent K^+^ channels and Na^+^-K^+^-ATPase (Roman et al. 2000). 20-HETE in vascular smooth muscle functions as a second messenger to promote depolarization, Ca^2+^ influx, and contraction of vascular smooth muscle that acts, in part, through protein kinase C (PKC) (Miyata and Roman 2005; Williams et al. 2010).

Ion channels are important determinants of vascular tone, as they control membrane potential and the intracellular Ca^2+^ concentration. Large conductance, Ca^2+^/voltage-sensitive K^+^ (BK) channels participate in this electromechanical coupling (Nelson et al. 1995; Brenner et al. 2000). BK channels are activated by membrane depolarization and increases in intracellular Ca^2+^. 20-HETE has been shown to inhibit BK channels in canine basilar artery (Obara et al. 2002) and rat renal arterioles (Zou et al. 1996). BK channels can also be regulated by phosphorylation and are targets of PKC, which reduces open probability (Zhou et al. 2009).

We have shown previously that activation of A_1_AR couples with the Cyp4a metabolite, 20-HETE and mediates contraction of the aortic smooth muscle through a pathway involving PKCα and/or p-ERK1/2. However, genetic ablation of the A_1_AR reduced the contractions in response to 20-HETE, in part, by reducing the expression of downstream signaling molecules (PKCα and p-ERK1/2) (Kunduri et al. 2013). To further understand the signaling transduction of A_1_AR and 20-HETE, we performed studies designed to test the hypothesis that activation of A_1_AR inhibits BK channels via a PKC-dependent mechanism.

## Materials and Methods

### Animals

A_1_AR knockout (A_1_KO) mice (originally obtained from Dr. Jurgen Schnermann, NIDDK, NIH) are on C57BL/6 background. A_1_KO mice were backcrossed four generations with C57BL/6 (wild type, WT); genotypes were confirmed by polymerase chain reaction. C57BL/6 (WT) mice (originally purchased from The Jackson Laboratory, Bar Harbor, ME) were bred in-house. Equal number of males and females of 14–18 weeks of age were used in our studies, as no gender differences were observed. The Institutional Animal Care and Use Committee of West Virginia University provided regulatory oversight and protocols followed guidelines set forth in *The Guide for the Care and Use of Laboratory Animals* (National Research Council, 2011). Mice had free access to food and water and were housed on a 12:12 h light–dark cycle. Mice were killed with an overdose of sodium pentobarbital (150 mg/kg ip) and aortae were quickly harvested into ice-cold physiological saline solution. Adipose and connective tissue were removed under the magnification of a dissecting microscope.

### Immunoblot analysis

Aortae from WT and A_1_KO mice were homogenized with 150 μL radio-immuno precipitation assay buffer containing (mmol/L) 20 Tris-HCl, 150 NaCl, 1 Na_2_EDTA, 1 EGTA, 2.5 sodium pyrophosphate,1 beta-glycerophosphate, and 1 Na_3_VO_4_; plus 1% NP-40, 1% sodium deoxycholate, and 1 μg/mL leupeptin. Samples were vortexed and then centrifuged for 10 min at 13,800 g at 4°C. Protein was measured using the Bradford dye procedure with bovine serum albumin as a standard (Bio-Rad Laboratories; Hercules, CA). The protein extract was divided into aliquots and stored at −80°C. Samples (25 μg of total protein) were loaded on slab gels (10% acrylamide; 1 mm thick), separated by Sodium dodecyl sulfate polyacrylamide gel electrophoresis, and transferred to nitrocellulose membranes (Hybond-ECL). Protein transfer was confirmed by visualization of prestained molecular weight markers (Bio-Rad). Membranes were blocked with 5% nonfat dry milk and incubated with primary antibody. A 1:5000 primary antibody dilution used for BK α and β1 subunits (Alomone laboratories, Jerusalem, Israel), while 1:10,000 dilutions were used for secondary antibody and β-actin.

### Electrophysiology

Wild type and A_1_KO mice aortae were digested in a physiological saline solution containing (mg/mL) 2 collagenase type-II, 1 soybean trypsin inhibitor, 1 bovine serum albumin, and 1 elastase for 30 min at 37°C. Single cells were liberated by passing the tissue through the tip of a fire-polished Pasteur pipette. The suspension was passed through a 100 μm nylon mesh and spun for 10 min at 10,000 g. The pellet was resuspended in low Ca^2+^ physiological saline solution and cells were stored on ice for use within 8 h. Cells were allowed to attach to glass coverslip, which was then transferred to the recording chamber. Solutions flowed into the recording chamber by gravity at a rate of 2-3 mL/min and the chamber had a volume of 0.2–0.3 mL. BK channel currents were recorded at room temperature from whole-cell patches as described previously (Asano et al. 2010). Bath solution contained (mmol/L) 135 NaCl, 5 KCl, 2 CaCl_2_, 1 MgCl_2_, 10 glucose, 10 HEPES free acid, and 5 Tris base; pH 7.4. Pipette solution contained (mmol/L) 140 KCl, 1 MgCl_2_, 1 EGTA and 0.281 CaCl_2_ (pCa 7), 10 HEPES, 1 Mg-ATP, 0.1 Na-GTP, and 5 Tris; pH 7.1. pClamp software and an Axopatch 200B amplifier were used (Molecular Devices; Sunnyvale, CA). Currents were low pass filtered at 1 kHz and digitized at 5 kHz.

### Statistics

Data are expressed as mean ± SEM from n number of mice, because the treatment level (i.e., genotype) is on a per mouse basis. For patch clamp experiments, that means results from all cells (≥3) from a single mouse aorta were averaged to represent *n* = 1. Current–voltage relationships were analyzed by two-way repeated measures analysis of variance (ANOVA). This was followed with Bonferroni post hoc test to determine where differences existed. When only two values were compared (e.g., BK subunit expression) an unpaired *t*-test was used. *P* < 0.05 was considered significant in all tests.

## Results

### Total BK current and BK subunit expression in WT and A_1_KO mice aortic myocytes

**Figure 1:**
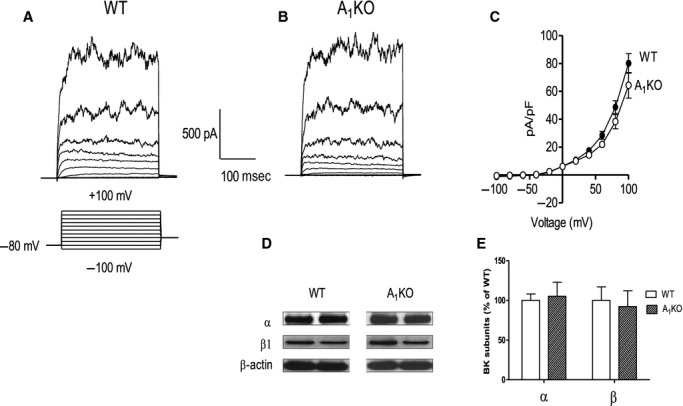
Whole-cell K^+^ current and BK channel subunit expression is similar in smooth muscle from wild type (WT) and A_1_KO mice. Representative traces of whole-cell K^+^ current in aortic smooth muscle cells from WT (A) and A_1_KO mice (B). The voltage template used to elicit the currents in this and subsequent figures is shown below the trace in A; cells were held at −80 mV and stepped from –100 to +100 mV in 20 mV increments. (C) Group data representing whole-cell K^+^ current in aortic smooth muscle cells from WT (*n* = 13) and A_1_KO (*n* = 20) mice. (D) Representative Western blots from mouse aortae for BK channel subunit expression relative to β -actin (α = 100 kDa; β 1 = 25 kDa; β –actin = 42 kDa). (E) Group data for BK α and β 1 subunit expression in the aortae of WT (*n* = 6) and A_1_KO (*n* = 6) mice. There were no differences between WT and A_1_KO mice in whole-cell K^+^ current or BK protein expression.

We performed whole-cell patch recordings on aortic smooth muscle cells from WT (Fig. 1A) and A_1_KO (Fig. 1B) mice; we observed no difference in BK current. That is, whole-cell K^+^ current in smooth muscle cells was indistinguishable between WT and A_1_KO mice. Currents were normalized to cell capacitance (i.e., current density). The group data are shown in Fig. 1C. BK α and β1 proteins were expressed in aortae from both WT and A_1_KO mice. BK α and β1 subunit proteins migrated at 100 and 25 kDa, respectively. There were no differences observed in the two protein levels between genotypes (Fig. 1D and E). Thus, the molecular (protein) and functional (current) expression of BK channels was similar in smooth muscle cells from WT and A_1_KO mice.

### Effect of 20-HETE on BK current in WT and A_1_KO mice aortic myocytes

**Figure 2:**
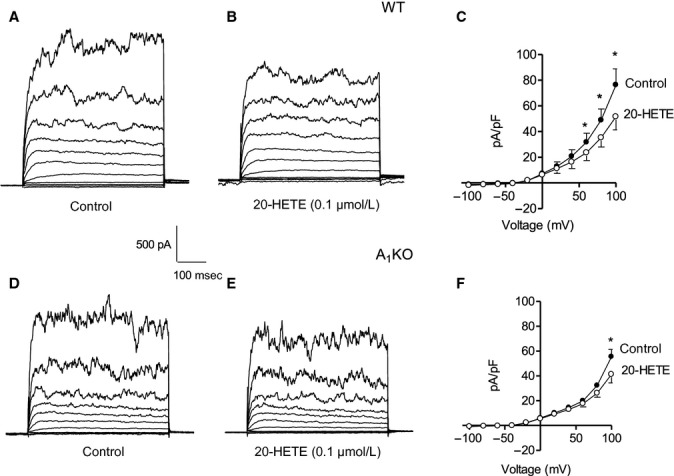
Effect of 20-HETE on BK current in wild type (WT) and A_1_KO aortic myocytes: Representative current traces are shown under control conditions (A) and with 0.1 μmol/L 20-HETE (B) in a smooth muscle cell from a WT mouse. The voltage template was the same as Figure 1. (C) Group data (*n* = 5) show the decrease in the BK current by 20-HETE in smooth muscle cells from WT mice. Representative traces are shown under control conditions (D) and with 0.1 μmol/L 20-HETE (E) for a smooth muscle cell from an A_1_KO mouse. (F) Group data (*n* = 5) show the decrease in BK current by 0.1 μmol/L 20-HETE in smooth muscle cells from A_1_KO mice. *P < 0.05 compared to the respective control.

To assess the reported inhibitory effect of 20-HETE on BK channels (Zou et al. 1996; Lange et al. 1997), whole-cell recordings were performed on WT and A_1_KO myocytes. We observed a decrease in the BK current in both WT (Fig. 2A and B) and A_1_KO (Fig. 2D and E) smooth muscle cells. Mean current density at +100 mV in WT under control conditions was 76.4 ± 12.5 pA/pF (*n* = 4); this was decreased to 51.6 ± 10.3 pA/pF by 20-HETE (Fig. 2C). In smooth muscle cells from WT mice, 20-HETE decreased current density 33 ± 7%. Similarly in smooth muscle cells from A_1_KO mice, mean current density was 55.6 ± 10.3 pA/pF (*n* = 4) and this was decreased to 41.4 ± 7 pA/pF by 20-HETE (Fig. 2F). Thus, in myocytes from A_1_KO mice, 20-HETE decreased current density 24 ± 11%.

### Effect of NECA on BK current in WT and A_1_KO mice aortic myocytes

**Figure 3:**
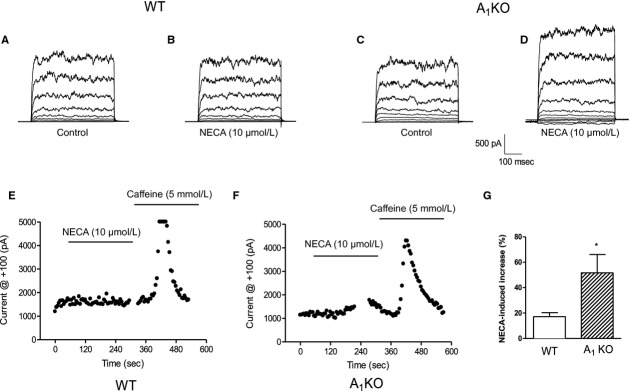
Effect of NECA on BK current in wild type (WT) and A_1_KO aortic myocytes: Representative currents under control conditions (A) and with 10 μmol/L NECA (B) in WT. The voltage template was the same as Figure 1. Representative currents under control conditions (C) and with 10 μmol/L NECA (D) in smooth muscle cells from A_1_KO mice. Data showing currents versus time for 10 μmol/L NECA and 5 mmol/L caffeine in smooth muscle cells from WT (E) and in A_1_KO (F) mice (blank areas in the time course represent where the protocol was stopped to perform voltage steps) (G) Group data show that NECA increases the BK current more in smooth muscle cells from A_1_KO mice compared to WT mice. *P < 0.05 for WT versus A_1_KO; *n* = 7–9.

Whole-cell patch recordings were made in WT and A_1_KO aortic myocytes to determine the effect of NECA on BK current. 5′–N–ethylcarboxamidoadenosine (NECA) is a nonselective adenosine receptor agonist and can activate multiple AR subtypes simultaneously. Whole-cell recordings showed prominent BK current in smooth muscle cells from WT and A_1_KO mice. Caffeine (5 mmol/L) was used as a positive control to release Ca^2+^ and increase BK current in both WT and A_1_KO aortic smooth muscle cells (Fig. 3E and F). There was very little change in the BK current in the WT aortic myocytes when stimulated with 10 μmol/L NECA (Fig. 3E). In contrast, the BK current in A_1_KO aortic myocytes was significantly increased by 10 μmol/L NECA (Fig. 3F). The time-dependent increase in BK current with 10 μmol/L NECA in A_1_KO smooth muscle cells was 52 ± 15% (*n* = 7); this was significantly higher than the response to NECA in smooth muscle cells from WT mice (17 ± 3%; *n* = 9; Fig. 3G). The disparate responses to NECA in smooth muscle cells from WT and A_1_KO mice suggest that multiple AR subtypes are simultaneously regulating BK channels. Thus, the next experiment was to determine the effect of an A_1_AR-specific agonist on BK channels in smooth muscle cells from WT and A_1_KO mice.

### Effect of CCPA on BK current in WT and A1KO mice aortic myocytes

**Figure 4:**
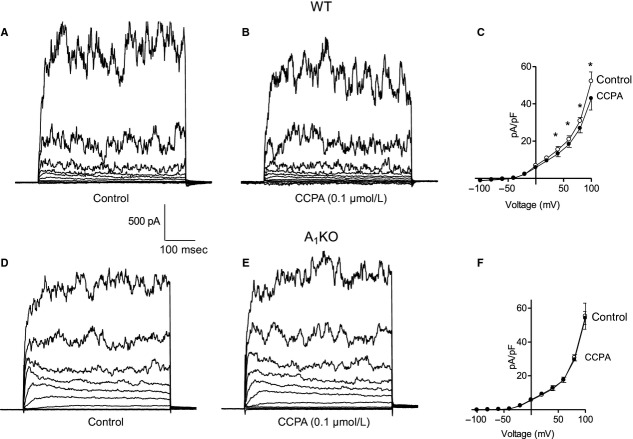
Effect of CCPA on BK current in wild type (WT) and A_1_KO aortic myocytes: Representative traces under control conditions (A) and with 0.1 μmol/L CCPA (B) in a smooth muscle cell from a WT mouse. The voltage template was the same as Figure 1. (C) Group data representing the decrease in the BK current by CCPA in the WT mice (*n* = 4). Representative traces show current under control conditions (D) and with 0.1 μmol/L CCPA (E) in a smooth muscle cell from an A_1_KO mouse. (F) Group data illustrate that there is no effect of CCPA on BK current in smooth muscle cells from A_1_KO mice. *P < 0.05 compared to untreated WT (*n* = 4).

2–chloro–N(6)-cyclopentyladenosine (CCPA; 100 nmol/L), an A_1_-selective agonist, decreased BK current in aortic smooth muscle cells from WT mice (Fig. 4A and B). The mean current density at +100 mV in WT was 52.1 ± 5.0 pA/pF (*n* = 4) and decreased with the application of CCPA to 42.8 ± 6.2 pA/pF (Fig. 4C). That is, CCPA decreased current density 19 ± 4% in smooth cells from WT mice. In contrast, CCPA had no effect on BK current in smooth muscle cells from A_1_KO mice (Fig. 4D and E). The mean current density at +100 mV in smooth muscle cells from A_1_KO mice was 55.2 ± 7.7 and 54.1 ± 3.9 pA/pF (*n* = 4) in the absence or presence of 100 nmol/L CCPA, respectively (Fig. 4F). That is, current density in the presence of CCPA was 98 ± 7% of control in smooth muscle cells from A_1_KO mice.

### Effect of PKCα inhibition on BK current in WT and A1KO mice aortic myocytes

**Figure 5:**
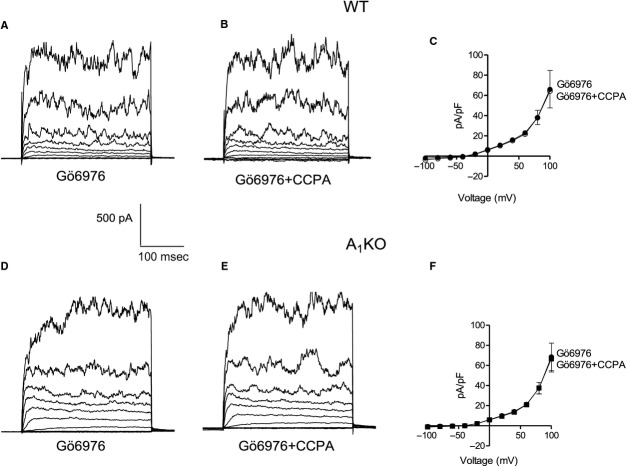
Effect of PKCα inhibitor, Gö6976 on BK current in wild type (WT) and A_1_KO aortic myocytes: Representative traces with 0.1 μmol/L Gö6976 (A) and with 0.1 μmol/L Gö6976 + 0.1 μmol/L CCPA (B) in a smooth muscle cell from a WT mouse. The voltage template was the same as Figure 1. (C) Group data demonstrate the effect of Gö6976 to prevent CCPA-induced inhibition of BK current in smooth muscle cells from WT mice (*n* = 4). Representative traces with 0.1 μmol/L Gö6976 (D) and with 0.1 μmol/L Gö6976 + 0.1 μmol/L CCPA (E) in a smooth muscle cell from an A_1_KO mouse. (F) Group data representing BK current in Gö6976-treated smooth muscle cells from A_1_KO mice (*n* = 4).

As shown previously (Ponnoth et al. 2012b; Kunduri et al. 2013), PKCα is downstream of A_1_AR activation, 20-HETE production, and mediates contraction of smooth muscle. We determined if inhibition of PKCα affected regulation of BK current by A_1_AR activation in smooth muscle cells from WT and A_1_KO mice. When PKCα was inhibited with Gö6976 (100 nmol/L) in WT smooth muscle cells, subsequent addition of CCPA (100 nmol/L) was no longer able to inhibit current (Fig. 5A and B; compare these to Fig. 4–A–C). In smooth muscle cells from WT mice, mean current density at +100 mV for Gö6976 was 65.9 ± 18.6 pA/pF versus 64.2 ± 16.6 pA/pF for CCPA + Gö6976 (Fig. 5C). That is, current density in the presence of CCPA was 99 ± 3% of control in smooth muscle cells from WT mice treated with Gö6976. There was no effect of CCPA on BK current in A_1_KO smooth muscle cells whether Gö6976 was present or not (Fig. 5D–F; note that this is a result similar to that shown in Fig. 4D–F). The mean current density at +100 mV in cells from A_1_KO mice for Gö6976 was 68.5 ± 13.6 pA/pF versus 66.6 ± 13.2 pA/pF for CCPA + Gö6976 (Fig. 5F). That is, current density in the presence of CCPA was 97 ± 1% of control in Gö6976-treated smooth muscle cells from A_1_KO mice.

## Discussion

We tested the hypothesis that activation of A_1_AR inhibits BK channels in aortic smooth muscle via a PKC-dependent mechanism. This hypothesis was based on previous studies indicating: (1) that A_1_AR stimulation is associated with 20-HETE production and activation of PKC (Ponnoth et al. 2012b; Kunduri et al. 2013) and (2) 20-HETE and PKC can inhibit BK channels (Zou et al. 1996; Nowicki et al. 1997; Schubert et al. 1999; Zhou et al. 2009). We performed whole-cell patch clamp and Western blot studies using aortic smooth muscle cells and aortae of WT and A_1_KO mice. No single-channel studies were done and this is a limitation of this study. Our major findings included the following: (1) There were no differences in whole-cell K^+^ current in aortic smooth muscle cells from WT and A_1_KO mice, nor were there any differences in the expression of pore-forming α or regulatory β1 subunit proteins. (2) Inhibition of BK current by 20-HETE was similar in aortic smooth muscle cells from WT and A_1_KO mice. (3) NECA, a nonselective AR agonist increased BK current in aortic smooth muscle cells from both WT and A_1_KO mice, but the increase was greater in smooth muscle cells from mice lacking the A_1_AR. (4) CCPA, an A_1_AR-selective agonist, inhibited BK current in smooth muscle cells from WT, but not A_1_KO, mice. (5) Inhibition of PKCα with Gö6976 abolished the effect of CCPA to inhibit BK current in smooth muscle cells from WT mice. Together, these data lead us to conclude that, in aortic smooth muscle, A_1_AR stimulation inhibits BK channel activity and that this occurs via a mechanism involving PKCα.

BK channels are ubiquitously expressed on the sarcolemma of vascular smooth muscles. BK channels are composed of pore-forming α subunits with or without regulatory β subunits. The β1 subunit, however, is commonly found in vascular smooth muscle (Nelson and Quayle 1995; Ledoux et al. 2006; Asano et al. 2010). The α subunit is the voltage- and Ca^2+^-sensitive pore, while β subunits can modify many characteristics including pharmacology and Ca^2+^-sensitivity. We observed no difference in the expression of α or β 1 BK subunits (Fig. 1), suggesting that the channels are equally expressed in aortic smooth muscle cells from WT and A_1_KO mice. Furthermore, there were no differences in BK current magnitude between WT and A_1_KO mice (Fig. 1). We have previously shown that adenosine A_1_ receptor-mediated smooth muscle contraction is dependent on Cyp4a using WT and A_1_AR knockout mice (Ponnoth et al. 2012b; Kunduri et al. 2013). 20-HETE has been shown to inhibit BK channels in rat renal arteriolar smooth muscle cells (Zou et al. 1996; Sun et al. 1999) and we wanted to investigate if A_1_AR-20-HETE-mediated pathway for smooth muscle contraction involved BK channels. We have observed similar results in whole-cell patch recordings (Fig. 2). That is, 20-HETE decreased BK current similarly in smooth muscle cells from both WT and A_1_KO mice (Fig. 2). In this study, we used only a single concentration of 20-HETE. This concentration was chosen based on our previous study (Kunduri et al. 2013) where we determined the concentration-dependence of aortic contraction to 20-HETE. The concentration of 20-HETE (0.1 μmol/L) used in this study is quite modest and it is in the range of 100 μmol/L (Zou et al. 1996; Sun et al. 1999) and 1 μmol/L (Zou et al. 1996) concentration that have been used by several others as well. 20-HETE is a potent vasoconstrictor as shown previously by significant contractions in the aortae of both WT and A_1_KO mice (Ponnoth et al. 2012b; Kunduri et al. 2013). 20-HETE activates PKC (Lange et al. 1997; Nowicki et al. 1997; Ponnoth et al. 2012b; Kunduri et al. 2013) and PKC may mediate contraction by inhibiting BK channel activity in rat cerebral arteries (Bonev and Nelson 1996), rabbit portal vein (Kitamura et al. 1992), canine basilar artery (Obara et al. 2002) and rat tail artery (Schubert et al. 1999). This inhibition depends on the sequential phosphorylation of two serines in the C-terminus of the BK α subunit (Zhou et al. 2009). In this study, when PKCα was antagonized with Gö6976, CCPA could no longer inhibit BK channel current (compare Figs. 4 and 5). This suggests that A_1_AR signaling through PKCα is negatively coupled to BK channels, perhaps by 20-HETE. The involvement of PKCα in 20-HETE-mediated inhibition of BK channels has been shown in canine basilar artery as well (Obara et al. 2002).

We cannot exclude the possibility that isoforms of PKC other than PKCα regulate BK channels. We relied on experiments with a single inhibitor (Gö6976) at a single concentration (100 nmol/L). However, our previously published data indicate that PKC α (alpha) is the isoform most abundant in mouse smooth muscle (Ansari et al. 2009). Other conventional isoforms were expressed, including beta (β) and gamma (γ), but at lower levels. With regard to conventional PKC isoforms, Gö6976 inhibits PKC α and β, but cannot differentiate between the two, as the IC_50_ values are close. However, PKC β is not likely to mediate this effect, as we demonstrated previously that activation of AR does not increase PKC β in the membrane fraction (Ansari et al. 2009). The amount of PKC in the membrane fraction is an indicator of enzyme activation. In contrast, after adenosine receptor activation, the amount of PKC α in the membrane fraction does increase (Ansari et al. 2009). The IC_50_ of Gö6976 for PKC γ appears to be at least 10 μM (Keenan et al. 1997). As for novel and atypical isoforms of PKC, Gö6976 does not inhibit PKC δ (delta), ε (epsilon), or ζ (zeta) at the concentration we used (IC_50_ > 3 μmol/L; [Martiny-Baron et al. 1993]).

The nonselective adenosine agonist NECA relaxes smooth muscle by acting on A_2_AR (Rump et al. 1999; Tawfik et al. 2005; Ponnoth et al. 2009), whereas the A_1_AR-selective agonist CCPA contracts smooth muscle (Ponnoth et al. 2012b; Kunduri et al. 2013). Activation of BK channels by A_2_AR could lead to membrane potential hyperpolarization and contribute to the relaxation of smooth muscle, whereas inhibition of BK channels by A_1_AR could cause depolarization and contribute to contraction. We observed that NECA increased BK current significantly in A_1_KO as compared to the WT (Fig. 3). This suggests that the increase in the BK current could be due to the absence of A_1_ and the nonselective action of NECA on other AR (e.g., A_2_AR) in the A_1_KO. Unpublished results from our lab suggest that A_2A_ receptor expression is upregulated in A_1_KO mice and this might also be a factor in the larger responses to NECA. However, studies investigating the effect of A_2A_ receptor signaling on BK current have not been performed and represent a limitation of this study. There is evidence showing that in the rat preglomerular vessels A_2A_AR, through EETs, activate BK channels mediate vasodilation (Carroll et al. 2006; Ray and Marshall 2006). Our own laboratory has shown evidence in the mouse aorta that A_2A_R mediates vasodilation through EETs via sarcolemmal K_ATP_ channels (Ponnoth et al. 2012a). In addition, it has been shown in the rats that A_3_AR restores vascular reactivity after hemorrhagic shock in a ryanodine receptor-mediated and BK channel-dependent pathway (Zhou et al. 2010). There have been no studies showing the interaction of A_2B_AR and BK channels. Furthermore, by using the A_1_ selective agonist CCPA we demonstrated a decrease in BK current in smooth muscle cells from WT mice, but no effect in smooth muscle cells from the A_1_KO mice. This is the first evidence in the literature showing that A_1_AR activation inhibits BK current. As A_1_AR is known to mediate contraction (Tawfik et al. 2005; Wang et al. 2010; Kunduri et al. 2013), we suggest this may be mediated by inhibition of BK channels. It should be noted, however, that there are reports of A_1_ activating K_ATP_ channels (Dart and Standen 1993) and linking to nitric oxide-dependent smooth muscle relaxation (Ray and Marshall 2006). The reasons for such differences are not readily apparent, but may perhaps be attributed to the vascular beds and species.

Reactive oxygen species (ROS) are known to play a role in modulating BK channel activity. Whether ROS activates or inhibits BK channel is debatable. For instance, studies have shown that hydrogen peroxide (H_2_O_2_) has negative effects (DiChiara and Reinhart 1997) and positive effects (Tang et al. 2001) on BK channel activation. However, ROS are generated by the activation of AR as shown by us (Sharifi Sanjani et al. 2013) and others (Narayan et al. 2001). Myocardial A_2A_ receptors via H_2_O_2_ couple with K_ATP_ channels in smooth muscle play a role in reactive hyperemia (Sharifi Sanjani et al. 2013). A_1_ receptors have been shown to attenuate myocardial stunning by reducing ROS formation by opening K_ATP_ channels (Narayan et al. 2001). There have been no studies showing a link between adenosine receptor activation, ROS generation, and BK channel activation. However, we can speculate that A_1_ receptor activation may involve ROS pathway in modulating BK channel activity, but further studies are needed to test this hypothesis.

There is no clear consensus on mean arterial pressure in WT and A_1_KO mice. Johansson et al. (2001) generated the first A_1_KO mouse line in 2001. These authors reported no change in blood pressure or heart rate. Unfortunately, the data were not shown and the paper does not indicate how measurements were made (Johansson et al. 2001). Brown et al. (2001) reported that MAP was increased in A_1_KO mice (same mice used by Johansson and colleagues from Fredholm's laboratory). Specifically, MAP in conscious mice was 85 ± 1 and 97 ± 2 mmHg for WT and A_1_KO mice, respectively (*P* < 0.05). In addition to changes in salt and water balance and renal hemodynamics, these data support the idea that A_1_ receptors couple to smooth muscle contraction and increases in total peripheral resistance (Brown et al. 2001). In contrast, Lee et al. 2012 reported that telemetered MAP in unstressed A_1_KO mice was indistinguishable from WT. Specifically, Lee et al. (2012) report that MAP is 120 ± 3 versus 117 ± 3 mmHg in WT and A_1_KO mice, respectively. When mice are made hypertensive with Ang II infusions, the blood pressure is less in A_1_KO mice, reinforcing the idea that A_1_ receptors couple to smooth muscle contraction and increased total peripheral resistance (Lee et al. 2012).

Adenosine, via multiple receptor subtypes, contracts and relaxes vascular smooth muscle through several mechanisms, including the regulation of K^+^ channels (Dart and Standen 1993). While adenosine-mediated increases in K_ATP_ channel activity are generally well accepted (Sharifi Sanjani et al. 2013), reports regarding the role of BK channels in adenosine-induced smooth muscle relaxation vary widely. In canine coronary arterioles, vasodilation in response to adenosine is inhibited by iberiotoxin (a very selective BK channel antagonist) (Cabell et al. 1994). Blocking BK channels inhibits vasodilation to 2-chloroadenosine in pig coronary arterioles (Borbouse et al. 2009); however, the role of BK channels in this response is abolished in pigs with metabolic syndrome (Borbouse et al. 2009). Thus, it could be pathology that explains why BK channels play no role in adenosine-induced vasodilation human coronary arterioles (Sato et al. 2005), as they are typically collected from patients with heart disease. Conversely, it may be that BK channels play little, if any role, in adenosine-induced vasodilation, as this has been reported in the majority of studies from pig coronary arterioles (Hein and Kuo 1999; Hein et al. 2001; Heaps and Bowles 2002). However, it cannot be ignored that BK channels are reported to contribute to adenosine-induced relaxation or vasodilation of rat cerebral arterioles (Paterno et al. 2012b), rabbit renal arteries (Rump et al. 1999), rat aortas (Ray and Marshall 2006), and rat preglomerular microvessels (Carroll et al. 2006). Furthermore, adenosine increases a Ca^2+^-dependent K^+^ current in smooth muscle cells from the rat mesenteric artery that may be mediated by BK channels (Li and Cheung 2000). At present, there is little consensus regarding the role of BK channels in adenosine-induced smooth muscle relaxation and very little data directly addressing whether adenosine increases BK current in smooth muscle cells isolated from those same arteries or arterioles.

Our lab has previously shown in the coronary artery smooth muscle cells that activation of A_1_AR leads to signaling via PLC-βIII, PKC-α leading to ERK1/2 phosphorylation. However, this study demonstrates that adenosine receptor signaling converges on BK channels in vascular smooth muscle. The study is novel in showing a relationship between A_1_AR; Cyp4a product, 20-HETE; PKC-α and BK channel in WT and A_1_KO aortic myocytes. However, this can be extrapolated to understand vasomotor responses in resistance vessels, but this will require direct investigation, outside the scope of this study. This conduit artery does not contribute to resistance that regulates blood flow, but results from this cell type provide insight into signaling mechanisms that may also be present in smaller arteries and arterioles. From our data, we conclude that A_1_AR signaling inhibits BK channels via 20-HETE and PKCα.
